# Supplementary material for: Effect of Skin Ion Channel TRPM8 Activation by Cold and Menthol on Thermoregulation and the Expression of Genes of Thermosensitive TRP Ion Channels in the Hypothalamus of Hypertensive Rats
Source: Int J Mol Sci. 2022 May 29;23(11):6088. doi: 10.3390/ijms23116088 (PMC9181123; doi:10.3390/ijms23116088)
Supplement: Supplementary file 1 [file ijms-23-06088-s001.zip › supplementary-Table S1.pdf]

Supplementary Materials:

Table S1. P-values for the comparisons of thermoregulatory responses represented at the Figure 1 and Figure 2. Thermoregulatory responses in hypertensive ISIAH rats subjected to the peripheral ion channel TRPM8 activation by menthol (application of a 1% suspension in saline to the skin) and control hypertensive ISIAH rats (application of saline) were compared by the t-test (Statistica 8 software package).

Parameters	Thermoregulatory responses	P value	
		Rapid cooling	Slow cooling
Latency periods of	constriction of ear skin vessels	0.107	0.718
	constriction of tail skin vessels	0.419	0.777
	increase of oxygen consumption	I phase 0.184<br>II phase 0.716	0.104
	increase of carbon dioxide excretion	I phase 0.135<br>II phase 0.784	0.142
	increase of muscle electrical activity	0.310	0.737
Threshold values of core body temperature for the initiations of	Constriction of ear skin vessels	0.270	0.653
	constriction of tail skin vessels	0.255	0.751
	increase of oxygen consumption	I phase 0.322<br>II phase 0.669	0.163
	increase of carbon dioxide excretion	I phase 0.421<br>II phase 0.669	0.197
	increase of muscle electrical activity	0.708	0.646
Threshold values of abdominal skin temperature for the initiations of	constriction of ear skin vessels	0.051	0.603
	constriction of tail skin vessels	0.125	0.976
	increase of oxygen consumption	I phase 0.580<br>II phase 0.052	0.219
	increase of carbon dioxide excretion	I phase 0.401<br>II phase 0.058	0.297
	increase of muscle electrical activity	0.416	0.147
Maximum changes in the parameters of heat production	increase of oxygen consumption	I phase 0.810<br>II phase 0.988	0.082
	increase of carbon dioxide excretion	I phase 0.538<br>II phase 0.614	0.133
	increase of muscle electrical activity	0.380	0.920
Maximum changes in heat loss parameters	constriction of ear skin vessels	0.347	0.229
	constriction of tail skin vessels	0.241	0.446
Maximum changes in the respiratory exchange ratio		0.420	0.472
