# Supplementary material for: Effect of Skin Ion Channel TRPM8 Activation by Cold and Menthol on Thermoregulation and the Expression of Genes of Thermosensitive TRP Ion Channels in the Hypothalamus of Hypertensive Rats
Source: Int J Mol Sci. 2022 May 29;23(11):6088. doi: 10.3390/ijms23116088 (PMC9181123; doi:10.3390/ijms23116088)
Supplement: Supplementary file 1 [file ijms-23-06088-s001.zip › supplementary-Table S2.pdf]

Supplementary Materials:

Table S2: The *Ppia* mRNA level (ng/µl) in the anterior and posterior parts of the hypothalamus in the rats of different experimental groups.

Thermal treatment	Pretreatment			
	saline		1% menthol	
	anterior hypothalamus data	posterior hypothalamus data	anterior hypothalamus data	posterior hypothalamus data
Thermoneutral conditions	13.4 ± 2.52	16.5 ± 2.12	19.7 ± 2.92	15.9 ± 2.10
Rapid cooling	16.5 ± 1.10	18.9 ± 3.12	19.5 ± 2.28	15.5 ± 2.18
Slow cooling	17.7 ± 2.32	18.8 ± 1.98	18.7 ± 2.01	15.2 ± 2.33

For factor "part of hypothalamus" $F_{(1,105)}<1$, $P>0.05$;
For factor "thermal treatment" $F_{(5,105)}<1$, $P>0.05$;
For the interaction of the factors "part of hypothalamus" and "thermal treatment" $F_{(5,105)}<1$, $P>0.05$.
